# Cumulative prognostic power of laminin genes in colorectal cancer

**DOI:** 10.1186/s12920-018-0332-3

**Published:** 2018-02-13

**Authors:** Vladimir V. Galatenko, Diana V. Maltseva, Alexey V. Galatenko, Sergey Rodin, Alexander G. Tonevitsky

**Affiliations:** 1 Moscow State University, Leninskie Gory, 119991 Moscow, Russia; 2 SRC Bioclinicum, Ugreshskaya str 2/85, 115088 Moscow, Russia; 3 Tauber Bioinformatics Research Center, University of Haifa, Mount Carmel, 3498838 Haifa, Israel; 4 Department of Medical Biochemistry and Biophysics, Karolinska Institutet, Scheelesväg 2, 17177 Stockholm, SE Sweden; 5 P. Hertsen Moscow Oncology Research Institute, National Center of Medical Radiological Research, Second Botkinsky Lane 3, 125284 Moscow, Russia

**Keywords:** Laminins, Cancer prognosis, Colorectal cancer

## Abstract

**Background:**

Laminins are a major family of extracellular matrix proteins and the main component of basement membranes. Laminins are involved in many if not all stages of cancer progression, and expression of laminin genes has prognostic value in various types of cancer, including colorectal. Only single laminin genes or components of a single laminin trimer with significant differential expression have been regarded as potential biomarkers to date.

**Results:**

Here we compared prognostic power of classifiers constructed from sets of laminin genes with that of any single laminin gene. The analysis showed that cumulative prognostic power of sets of laminin genes was higher and was achieved already with pairs and triples of the genes. Interestingly, components of the pairs and the triples did not belong to any known laminin trimer, but, taken together with the gene weights, suggested higher *LAMA4/LAMA5* expression ratio in patients with poor prognosis.

**Conclusions:**

Analysis of the laminin expression profile rather than expression of the single genes or components of laminin trimers is useful for colorectal cancer prognosis in patients. High *LAMA4/LAMA5* ratio is associated with increased permeability of basement membranes suggesting that basement membranes produced by colorectal tumors might be an important hindrance to their own dissemination in patients.

## Background

Laminins are secreted, multidomain heterotrimeric proteins that consist of one α, one β, and one γ chains [1]. Five laminin α, four β, and three γ chains, which form not less than 16 different trimers, have been discovered in humans to date [2]. Unlike the majority of extracellular matrix proteins, laminins exhibit a certain degree of tissue and temporal specific expression [3]. As the main component of basement membranes (BMs), laminins affect various properties of BMs, for instance their permeability to cells.

Laminins are significantly involved in survival and proliferation of tumors and cancer cells, angiogenesis, migration and breaching of BMs by cancer cells, development of pre-metastatic niches at distant organs and many other stages of cancer progression (recently reviewed in [4]). A plethora of studies has demonstrated prognostic value of laminins in a range of cancers, including colorectal [4, 5]. However, these studies were focused only on expression of single laminin genes [6–8] or, rarely, components of a single laminin trimer [9]. Meanwhile, it has been shown that prognostic power of a set of genes can be higher that a sum of prognostic powers of the individual genes from the set [10]. Here we sought to identify sets of laminins with higher cumulative prognostic power than single laminin chains in colorectal cancer.

## Methods

### Estimation of prognostic power of laminin genes

Estimation of cumulative prognostic power of laminin genes was performed according to a previously described method [10]. In short, the estimation included construction classifiers based on expression levels of laminin genes (all laminin genes or subsets of laminin genes), filtering out low-quality classifiers, and evaluation of quality of these classifiers. Classifier construction was performed using soft-margin support vector machine (SVM) [11] with linear kernel applied to a training dataset (see Microarray datasets). At the filtration stage quality of the classifiers was evaluated independently on two filtering datasets (see Microarray datasets). The area under receiver operating characteristic (ROC) curve (AUC – area under ROC curve) was used as the main characteristic of the classifiers quality. A classifier passed filtration stage if AUC values were not less than 0.6 for the training dataset and not less than 0.55 for each filtration dataset. Final quality evaluation was performed on a testing dataset (see Microarray datasets) that was a totally independent dataset and contained no data intersections with the training or the filtering datasets.

### Microarray datasets

The utilized meta-set included GSE39582 [12] dataset that was used for training; GSE37892 [13] and GSE17536 [14, 15] datasets used for filtration; and GSE14333 [16] dataset used for validation of the results (testing dataset). The analyzed datasets (which were all based on Affymetrix Human Genome U133 Plus 2.0 Arrays) were jointly preprocessed using Robust Multi-array Average (RMA) method [17]. The patients with unknown recurrence status or with recurrence during the first month of follow-up were excluded from the analysis. Information on the number of patients in each dataset is presented in Table 1.

**Table 1:** Number of patients in datasets

	*Number of patients*		
	*Total number*	Without recurrence and time of follow-up not less than 4 years	With recurrence within 3 years
GSE39582	519	245	124
GSE37892	129	48	32
GSE17536	144	58	31
GSE14333	225	85	41

Only patients with a recurrence during the first 3 years and patients without a diagnosed recurrence and at least 4 years of follow-up were used for training and filtration as well as for construction of the ROC-curves and calculation of AUC for the testing dataset. Kaplan-Meier curves were plotted based on the complete testing dataset, including information on patients without a recurrence but time of follow-up less than 4 years and patients with recurrence but at least 3 years recurrence-free survival.

If the array contained several probes for a laminin chain, only one probe (with the highest intensity of the signal) was taken for the analysis. Laminin genes *LAMB4* and *LAMC3* were excluded from the analysis, because they exhibited stably low expression (95-th percentile of log_2_ expression level less than 7).

### Limitations of the study

All the data analyzed within the study have been acquired using Affymetrix Human Genome U133 Plus 2.0 Arrays and have not been confirmed by an independent method. Although generally reliable, problems with sensitivity, accuracy, and specificity have been reported for microarray results before [18]. Thus, specificity of microarray data may be compromised by non-specific hybridization that is a binding of not completely complimentary sequences to each other. However, it is worth noting that in a study [10] a gene signature identified using the same method based on similar microarrays (Affymetrix Human Genome U133A) successfully passed validation on a RNA-sequencing dataset. Another issue is possible discrepancies between expression of mRNAs and amounts of corresponding proteins due to differences in translation rates, half-lives of proteins, etc.

Irrespective of expression profiling technology, the method we used in this study for assessing prognostic power of gene sets and for finding small gene sets with the highest prognostic power has certain limitations. Lacking formal statistical background for associating *p*-values with the resulting prognosis reliability (expressed, e.g., in terms of AUC), it requires a large meta-set of samples (at least 120 samples in each group), and statistical significance can be assessed mainly using Monte Carlo resampling approaches. These limitations are not restrictive for this study.

### Statistics

Statistical significance of divergence of Kaplan-Meier survival curves was assessed using log-rank test. The presented *p*-values are two-tailed.

The statistical significance of the prognostic power of gene sets was assessed using Monte Carlo label permutation approach. Three permutation strategies were applied: labels permutation only in a training dataset, only in a testing dataset, and in both. Considering all permutations equiprobable, 10,000 permutations were generated for each strategy, and *p*-values were estimated based on AUC for the testing dataset in a standard way [19]. The highest (i.e., the most conservative) of three resulting values was reported.

## Results

**Fig. 1:**
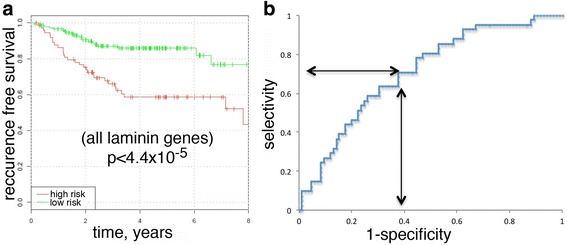
Cumulative prognostic power of all laminin chains for the testing dataset. (**a**) Kaplan-Meier curves. (**b**) ROC-curve

To assess prognostic power of the entire laminin family, we constructed a classifier based on all the laminin genes. The AUC value for the resulting classifier was equal to 0.710 for the testing dataset, and Monte Carlo label permutation approach confirmed the statistical significance of this AUC value (*p*-value < 2.7 × 10^− 3^). Consistently, a divergence of Kaplan-Meier curves for this classifier (Fig. 1a) was also highly significant (*P* = 4.4 × 10^− 5^). At the sensitivity level of 70% the specificity on the testing dataset noticeably exceeded 60% (Fig. 1b).

*LAMA4*, *LAMC1*, and *LAMC2* genes had the highest weights in the constructed classifier (Table 2). Higher expression of each of these genes was associated with an increase in the risk of colorectal cancer recurrence. Weights of the rest of the laminin genes were lower, but aggregated weight was comparable with that of the each of the three.

**Table 2:** Weights of genes in the classifier based on all laminin chains. A positive value of the weight indicates that higher expression of the gene is associated with higher risk of recurrence. A negative weight indicates that higher expression of the gene is associated with lower risk of recurrence

Gene	Microarray identifiers	Weight
*LAMA1*	227048_at	−0.024
*LAMA2*	213519_s_at	−0.104
*LAMA3*	203726_s_at	0.010
*LAMA4*	202202_s_at	0.381
*LAMA5*	210150_s_at	−0.111
*LAMB1*	201505_at	−0.051
*LAMB2*	216264_s_at	0.011
*LAMB3*	209270_at	−0.049
*LAMC1*	200771_at	0.306
*LAMC2*	202267_at	0.324

**Fig. 2:**
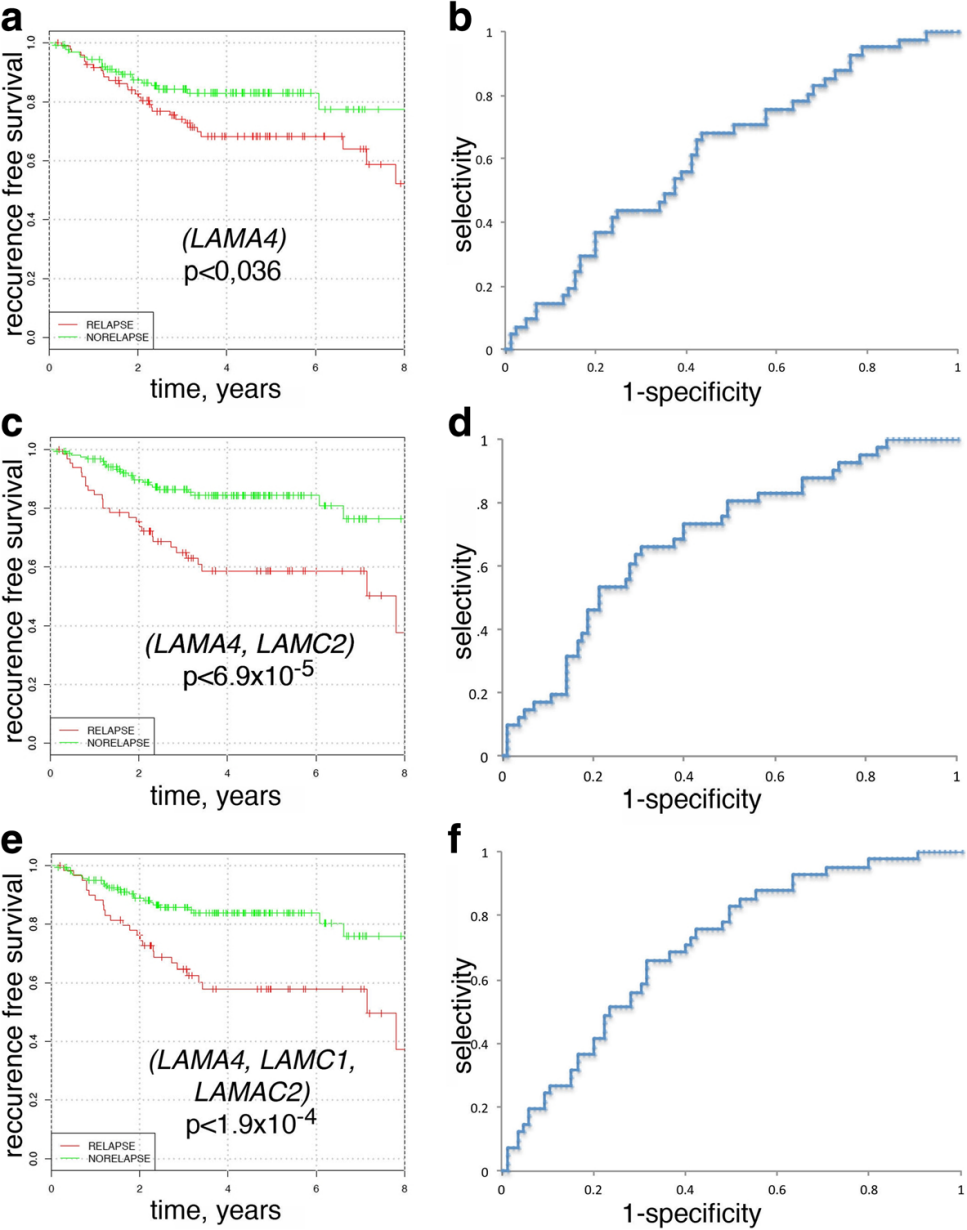
Cumulative prognostic power of single, pairs, and triples of laminin chains for the testing dataset. Kaplan-Meier curves and ROC-curves for *LAMA4* (**a** and **b**, respectively); for (*LAMA4*, *LAMC2*) pair (**c** and **d**); and for (*LAMA4*, *LAMC1*, *LAMC2*) triple (**e** and **f**)

To determine whether the set of laminin genes could be reduced to *LAMA4*, *LAMC1*, and *LAMC2* or even to a smaller subset of the genes without a significant reduction of prognostic power, we performed the analysis for the subsets of the laminin genes, starting from single laminin chains (Fig. 2a-f). Surprisingly, only *LAMA4* gene and not *LAMC2* passed filtration threshold (see Materials and Methods). But even for *LAMA4*, the quality of the classifier was much lower than that for the classifier comprising all laminin genes. The AUC value of *LAMA4* classifier was equal to 0.62 for the testing dataset and a *p*-value indicating statistical significance of Kaplan-Meier curves’ divergence was equal to 0.036 (Fig. 2a and b).

Analysis of prognostic power of pairs of laminin genes revealed that any pair of the genes from *LAMA4*, *LAMC1*, and *LAMC2* provides a classifier, which successfully passes filtration stage. Classifiers based on (*LAMA4*, *LAMC1*), (*LAMA4*, *LAMC2*), and (*LAMC1*, *LAMC2*) had the AUC values equal to 0.643, 0.692, and 0.726, respectively, for the testing dataset and the *p*-value associated with Kaplan-Meier curves’ divergence equal to 0.0057, 6.9 × 10^− 5^, and 0.0016, respectively. Therefore, prognostic power of (*LAMA4*, *LAMC2*) and (*LAMC1*, *LAMC2*) gene pairs was comparable with that of all laminin genes (Fig. 2c and d). Surprisingly, there were several other pairs containing other than *LAMA4*, *LAMC1*, and *LAMC2* genes with similar prognostic power in colorectal cancer. For instance, a classifier based on (*LAMA3*, *LAMC1*) had the AUC value 0.688 and the *P*-value 3.3 × 10^− 5^. Analysis of triples of laminin genes also revealed that other than (*LAMA4*, *LAMC1*, *LAMC2*) triples had similar prognostic power, for instance (*LAMA5*, *LAMC1*, *LAMC2*) and (*LAMA1*, *LAMA4*, *LAMC2*). In these triples, consistently with the classifier based on all laminin genes, higher expression of *LAMA1* and *LAMA5* was associated with lower risk of the recurrence.

## Discussion

As expected, cumulative prognostic power of all laminin chains was higher than that of any single chain, but, interestingly, the prognostic power of all laminins was achieved already with some pairs and triples of the genes. *LAMA4*, *LAMC1*, and *LAMC2* genes had the highest weights in the classifier based on all laminin chains. Several independent groups have demonstrated prognostic value of *LAMC2* in colorectal cancer before [7, 8, 20]. To our knowledge, there is no published data on prognostic value of *LAMA4* and *LAMC1* in colorectal cancer, but α4-containing laminins affect migration [21] and invasion [22] of various cancer cells and expression of *LAMC1* is associated with cancer progression in uterine carcinoma [23] and glioblastoma [24].

Unlike previously identified laminin biomarkers, the prognostic gene signatures found in this study also suggest a specific molecular mechanism that might be involved in progression of colorectal cancer. Indeed, all the mentioned above predictive classifiers based on the triples of laminin genes might suggest an increased permeability of cancer BMs in patients with higher risk of colorectal cancer recurrence. High risk of recurrence was associated with increased expression of α4 or decreased expression of α5 laminin chains. Laminin polymer of the vascular BMs, which is an important component of the vascular wall, is primary made of α4- and α5-containing laminins [25]. It has been shown that leukocytes have a reduced ability to penetrate the vessel walls that are depleted of α4-containing laminins [25, 26]. Since molecular mechanisms of diapedesis through the vessel wall for leukocytes and cancer cells are similar, we hypothesize that increased ratio of expression of α4-containing to α5-containing laminins might lead to increased permeability of cancer BMs for cancer cells facilitating their detachment from the primary tumor. Therefore, BMs produced by cancer cells might be an important hindrance to dissemination of colorectal cancer. Although the hypothesis needs an experimental confirmation, it might shed new light on molecular mechanisms of colorectal cancer progression in patients.

## Conclusions

We confirmed that laminins have a significant prognostic value in colorectal cancer. Prognostic power of any single laminin chain was lower than cumulative prognostic power of certain sets of laminin genes. Already some pairs and triples of laminin genes were sufficient to achieve the prognostic power of the entire laminin family. Detailed analysis of those pair and triples as well as genes weights in the classifiers based on laminin genes suggested that basement membranes produced by colorectal tumors might be an important hindrance to their own dissemination in patients.

## Abbreviations

AUC: Area under ROC curve
BMs: Basement membranes
RMA: Robust Multi-array Average
ROC: Receiver operating characteristic
SVM: Support vector machine
